# Low-temperature crystal structure of the unconventional spin-triplet superconductor UTe_2_ from single-crystal neutron diffraction

**DOI:** 10.1107/S2052520619016950

**Published:** 2020-01-29

**Authors:** Vladimir Hutanu, Hao Deng, Sheng Ran, Wesley T. Fuhrman, Henrik Thoma, Nicholas P. Butch

**Affiliations:** a Institute of Crystallography, RWTH Aachen University and Jülich Centre for Neutron Science (JCNS) at Heinz Maier-Leibnitz Zentrum (MLZ), Lichtenbergstrasse 1, Garching, 85748, Germany; b NIST Center for Neutron Research, National Institute of Standards and Technology, Gaithersburg, MD 20899, USA; c Center for Nanophysics and Advanced Materials, Department of Physics, University of Maryland, College Park, MD 20742, USA; d Jülich Centre for Neutron Science (JCNS) at Heinz Maier-Leibnitz Zentrum (MLZ), Lichtenbergstrasse 1, Garching, 85748, Germany

**Keywords:** crystal structure, single-crystal neutron diffraction, phase transition, unconventional superconductivity, magnetic order, UTe_2_

## Abstract

The crystal structure of a new superconductor UTe_2_ has been investigated using single-crystal neutron diffraction for the first time at the low temperature (LT) of 2.7 K, just above the superconducting transition temperature of ∼1.6 K, in order to clarify whether the orthorhombic structure of type *Immm* (No. 71), reported for the room-temperature (RT) structure persists down to the superconducting phase and can be considered as a parent symmetry for the development of spin-triplet superconductivity. In contrast to the previously reported phase transition at about 100 K [Stöwe (1996). *J. Solid State Chem*. **127**, 202–210], our high-precision LT neutron diffraction data show that the body-centred RT symmetry is indeed maintained down to 2.7 K. No sign of a structural change from RT down to 2.7 K was observed. The most significant change depending on temperature was observed for the U ion position and the U–U distance along the *c* direction, implying its potential importance as a magnetic interaction path. No magnetic order could be deduced from the neutron diffraction data refinement at 2.7 K, consistent with bulk magnetometry. Assuming normal thermal evolution of the lattice parameters, moderately large linear thermal expansion coefficients of about α = 2.8 (7) × 10^−5^ K^−1^ are estimated.

## Abstract 2

In UTe_2_ the body-centred space group *Immm* (No. 71) persists down to the very low temperatures, where the unconventional superconductivity develops.

## 1. Introduction

Very recently, unconventional spin-triplet superconductivity has been reported in UTe_2_ below 1.6 K (Ran, Eckberg *et al.*, 2019 ▸; Aoki *et al.*, 2019 ▸). It was proposed that it belongs to the family of U-based unconventional ferromagnetic (FM) superconductors as a paramagnetic end-member of this series, where spin fluctuations without an ordered magnetic moment (Sundar *et al.*, 2019 ▸; Metz *et al.*, 2019 ▸) play a major role in Cooper pairing. Moreover, observed superconductivity seems to survive the application of a very strong magnetic field, contrary to any intuitive expectation and even exhibits a separate re-entrant superconducting phase between 45 T and 60 T (Ran, Liu *et al.*, 2019 ▸; Knebel *et al.*, 2019 ▸). The structural parameters of UTe_2_ at low temperature (LT) are an important prerequisite for further studies and understanding of the intriguing phenomenon of FM superconductivity, especially by theoretical modelling and *ab* *initio* calculations (Jiao *et al.*, 2019 ▸; Xu *et al.*, 2019 ▸; Ishizuka *et al.*, 2019 ▸; Shick & Pickett, 2019 ▸). To the best of our knowledge, the LT structure of UTe_2_ has not been reported previously. In this regard the question occurs, whether the known room-temperature (RT) crystal structure continues to persist down to the very LT of the superconducting transition and how much the structural parameters change.

Historically, the crystal structure of UTe_2_ at RT was reported inconsistently (Ferro, 1954 ▸; Haneveld & Jellinek, 1970 ▸, 1969 ▸). The first exhaustive crystallographic study using single-crystal X-ray diffraction carried out in the late 1980s (Beck & Dausch, 1988 ▸) confirmed the ortho­rhombic space group *Immm* and reported precise crystallographic details, proved later by Ikeda *et al.* (2006 ▸). The only temperature-dependent study on the crystal structure of UTe_2_ were performed by Stöwe (Stöwe *et al.*, 1997 ▸; Stöwe, 1996 ▸). These authors studied the thermal evolution of the crystal structure in UTe_2_ by X-ray powder diffraction in the temperature range 300–10 K and by single-crystal X-ray diffraction in the temperature range 573–118 K, respectively. The single-crystal data did not reveal any anomalies; however, powder diffraction showed a clear phase transition between 110 and 92 K where a significant change in all three lattice parameters occurs. The structure below 90 K was not observed to change down to 10 K, demonstrating unexpectedly temperature-independent lattice parameters, although at 10 K the sample was determined to be in a metastable state probably due to heating by the X-ray beam. The observed phase transition at about 100 K is claimed to be robust and reversible in temperature. Below 23 K the occurrence of new additional reflections is reported. Unfortunately, the structure of this LT phase could not be determined by the used X-ray powder method (Stöwe, 1996 ▸). Ran, Eckberg *et al.* (2019 ▸) performed the first attempt to determine the crystal structure of UTe_2_ at LT (5 K) using cold neutron powder diffraction. Typically, neutron beams do not heat the sample. Because of their high-penetration ability through the metallic cryostat walls, neutron diffraction is the method commonly used at very LT. No additional peaks in the LT neutron powder diffraction pattern, compared with the RT X-ray one, were observed in discrepancy with the previous findings; however, no structural refinement was performed (Ran, Eckberg *et al.*, 2019 ▸).

Two possible scenarios are applicable. Either the structure does indeed not change, and the lattice parameter discontinuity reported by Stöwe (1996 ▸) may be just a result of some experimental artefacts, or the powder method does not distinguish between two possibly close symmetries. In order to check these scenarios and rule out possible ambiguity in structural determination and to provide structural details on UTe_2_ close to the superconducting phase transition, we performed a single-crystal neutron diffraction experiment at LT. The obtained data were refined starting from the RT orthorhombic structural model (space group *Immm*) and following the possible highest group–subgroup symmetry lowering relation paths.

## 2. Experimental

High-quality single crystals of UTe_2_ with typical size up to 3 mm × 3 mm × 1 mm and mass of ∼20–60 mg were obtained by the chemical vapour transport method. A crystal from the same growth batch as those described by Ran, Eckberg *et al.* (2019 ▸) was used for the present study. Single-crystal neutron diffraction was performed on the POLI diffractometer (Hutanu, 2015 ▸; Huţanu *et al.*, 2009 ▸) at the Heinz-Maier-Leibnitz Zentrum in Germany. A short neutron wavelength of 0.9 Å was employed in order to reduce potential parasitic effects of absorption and extinction. The corrected integrated intensities of the measured reflections were obtained with the *DAVINCI* program (Sazonov, 2015 ▸). The refinement of the structural parameters were performed with the program *JANA2006* (Petricek *et al.*, 2014 ▸). The sample was cooled down to 2.7 K (1 K above the superconducting transition in the normal-conducting state) and centred in the vertical position. A temperature control of better than ± 0.1 K was achieved. In a preliminary quick test-scan a total of 448 Bragg reflections with sinθ/λ ≤ 0.63 Å^−1^ were collected. As a result of the test, 327 reflections satisfying the criterion *I*_max_ > 1.75*I*_background_ were selected for a further detailed measurement. The selected peaks were individually pre-centred and carefully measured by an omega scan. After the profile analysis of the measured peaks, a total of 298 individually-centred Bragg reflections satisfying the criterion *I* > 10σ(*I*) were used for the refinement.

Experimental details are summarized in Table 1 ▸. The lattice parameters at 2.7 K noted there were obtained by refinement of the orientation matrix using the angular positions of the strongest 200 centred peaks and fixed known offsets for the instrument axes. In the following, the LT values are compared to RT data (Beck & Dausch, 1988 ▸; Ikeda *et al.*, 2006 ▸). The lattice shrinks upon cooling relatively homogeneously in all directions: Δ*a*/*a* = 0.87 (13)%, Δ*b*/*b* = 0.62 (16)%, Δ*c*/*c* = 0.96 (14)%. This corresponds to about 2.5% volume reduction and an approximate average linear coefficient of thermal expansion of α ≃2.8 (7) × 10^−5^ K^−1^. This value is in good agreement with the thermal evolution of lattice parameters determined from X-ray powder diffraction at higher temperatures (Stöwe, 1996 ▸).

## 3. Symmetry analysis, results and discussions

Starting from the orthorhombic structural model of space group *Immm* at RT, assuming a one-step symmetry lowering at an intermediate temperature [in agreement with findings by Stöwe (1996 ▸)] and considering the group–subgroup relationship, a few possible subgroups can be identified as potential candidate structures for the refinement of our LT data. Those subgroups belong to two main types: *translationengleiche*, which keep the same translation behaviour and *klassen­gleiche*, which preserve the symmetry class. Table 2 ▸ shows a list of the possible maximal subgroups of space group *Immm.* The violation of the general extinction condition for Bragg reflections with indices *h* + *k* + *l* = 2*n* + 1 would mean a loss of the body-centering. We performed a focused search for these Bragg reflections with *h* + *k* + *l* = 2*n* + 1 [*e.g.* (201), (210), (300), (030), (120)] and did not observe them up to level of less than < 5 × 10^−3^ of the intensity of the allowed reflections with *h* + *k* + *l* = 2*n*. It is worth mentioning that this is just the threshold where the parasitic effects, such as higher-order wavelength contamination or Renninger scattering, start to be significant. The absence of those peaks means that the body-centering is preserved and, thus, the *klassengleiche* subgroups could be ruled out. From the remaining *translation­engleichen* subgroups one is monoclinic (No. 12). By transformation to a monoclinic lattice, peak splitting may occur. Visual inspection of the over 400 measured reflection profiles did not reveal any splitting, suggesting that the lattice remains orthorhombic within the resolution of our experiment. The general extinction condition for the monoclinic space group No. 12 is *h* + *k* = 2*n* + 1. We observed many (more than 90 among the 300 peaks measured with highest precision) strong reflections with *h* + *k* = 2*n* + 1 violating this condition; thus, the monoclinic space group No. 12 is also ruled out.

**Figure 1:**
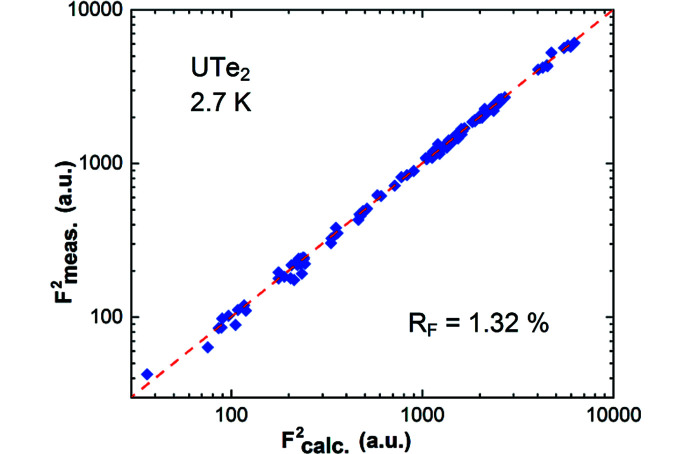
Quality of the diffraction data refinement for the nuclear structure of UTe_2_ at 2.7 K in space group *Immm*. The experimental measured structure factors (*F* ^2^_meas_) are plotted against the calculated ones (*F* ^2^_calc_) on a logarithmic scale for better visualization of the weak reflections.

The only remaining maximal space group for the refinement compatible with the observed extinctions are orthorhombic: *Immm* (No. 71), *Imm*2 (No. 44) and *I*222 (No. 23). It is worth mentioning that the powder diffraction study performed by Ran, Eckberg *et al.* (2019 ▸) would not be able to distinguish between them. It addition, other (non-maximal subgroup) orthorhombic space groups which satisfy the observed extinction symbol (*e.g.* *I*2_1_2_1_2_1_ No. 24) exist. However, in order to reach those symmetries starting from the parent space group No. 71, multiple phase transformations (at least three or more) would be necessary. Moreover, the body-centring would be lost in the required intermediate phases. As no experimental evidence exists either for such multiple phase transitions or for body-centring lost, we did not consider the refinement using those models. We performed the refinement of our single-crystal data in all mentioned space groups (Table 3 ▸). The starting parameters for the least-squares refinement were obtained from the RT structure determined by Beck & Dausch (1988 ▸). The results are very similar and show the high quality of the fit for all space groups. The larger number of free parameters used for space group *Imm*2 in comparison to the other two candidates does not improve the fit quality. Thus, *Imm*2 could be discarded. Among the remaining two, *Immm* has a higher symmetry than *I*222 and should be considered as a proper structure of UTe_2_ at 2.7 K. Thus, our single-crystal diffraction results show no evidence for the symmetry lowering at LT compared to the RT structure. Using space group *Immm* and averaging symmetry-equivalent peaks, precise structural parameters of UTe_2_ at 2.7 K were refined. Resulting atomic coordinates and both the isotropic and anisotropic atomic displacement parameters (ADPs) (*U*_iso_, *U*_*ij*_) are presented in Table 4 ▸. Full details of the refinement, including bond lengths and angles, are provided in the deposited crystallographic information file (CIF) (ICSD http://www.fiz-karlsruhe.de, No. 1972889). The quality of the fit is shown in the Fig. 1 ▸. The high quality of the fit for our LT neutron data using the RT structural model (with adjusted parameters) may be linked to careful data collection on the one hand and perfectly matching structural model on the other.

**Figure 2:**
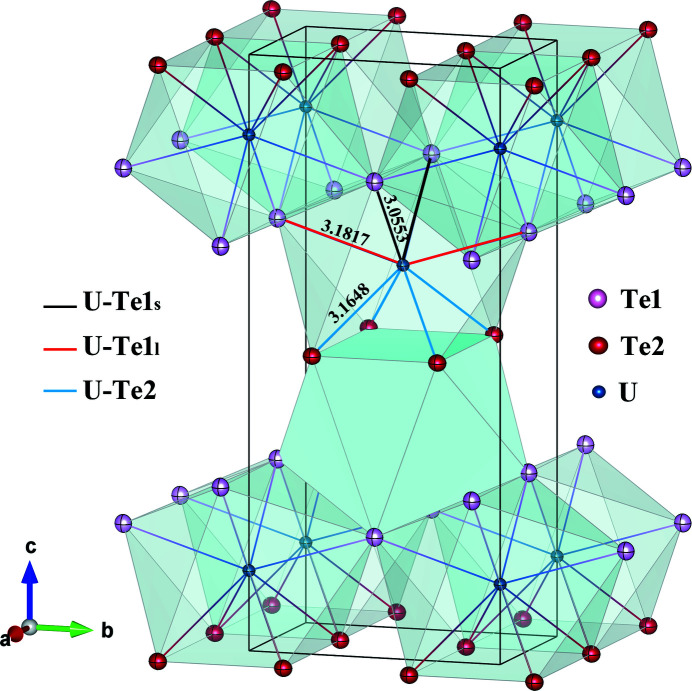
The first coordination-sphere polyhedron of U (cation) by neighbouring Te (anions) in UTe_2_ at 2.7 K. The bond lengths given are in Å. *VESTA* software (Momma & Izumi, 2011 ▸) was used for visualization. The atomic positions are shown by anisotropic displacement parameters with 99% probability.

Fig. 2 ▸ shows the perspective view of the UTe_2_ crystal structure. The positions of the atoms are shown by the ellipsoids of the refined ADPs with probability as high as 99%. The shape and absolute values of the ADPs reflect both atomic motion and possible static displacive disorder and, therefore, are often used as a hint to the potential symmetry lowering or structural distortions (Schweiss *et al.*, 1994 ▸). Small, almost spherical displacement parameters, showing no significant elongations, are observed for Te atoms independent of the Wyckoff position. Even smaller parameters are refined for U atoms at LT giving no indication about potential distortion of the assumed structure.

**Figure 3:**
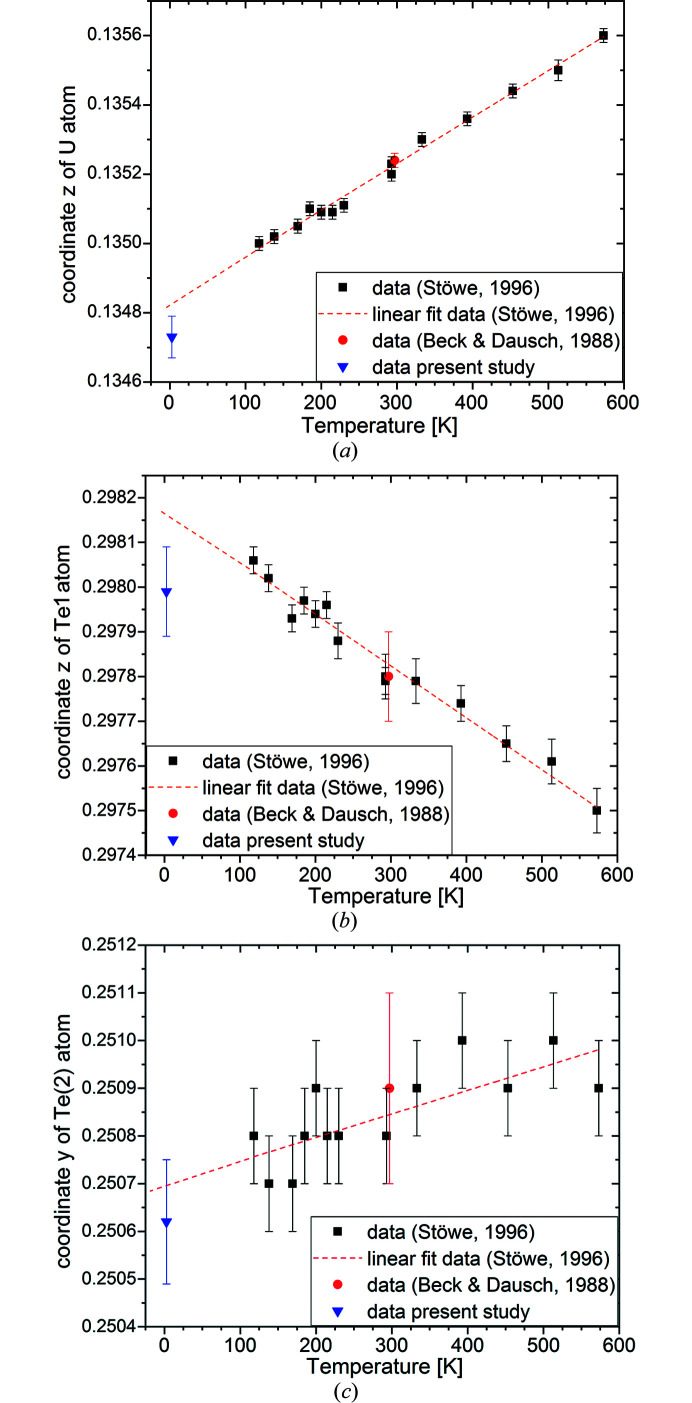
Comparison of the refined in the space group *Immm* general atomic coordinates for UTe2 at 2.7 K to the literature data at higher temperatures: (*a*) *z*(U), (*b*) *z*(Te1), (*c*) *y*(Te2).

The atomic coordinates *z*(U), *z*(Te1) and *y*(Te2) at LT shown in Table 4 ▸ were compared with those obtained from the single-crystal X-ray diffraction in the temperature range 573–118 K (Stöwe, 1996 ▸). Linear extrapolation of the large thermal evolution region (Stöwe, 1996 ▸) down to zero temperature reproduces reasonably well (within one to two sigma error bars) our results for 2.7 K (Fig. 3 ▸). The slightly lower values observed for *z*(U) and *z*(Te1) compared to the ones resulting from the linear trend extrapolation of the literature data assuming no phase transition, show the opposite thermal behaviour. *z*(U) decreases by temperature lowering and the found coordinate is even lower than the extrapolated value confirming or somehow overperforming the trend. On the other hand, the *z*(Te1) value increases with temperature lowering but the experimental Te1 coordinate is lower than extrapolated one, thus somehow underperforming the high-temperature data trend. The overall deviation along *z* of the group U–Te1 maintains thus a near-linear behaviour down to very low temperatures. Taking into account that the observed deviations are small, our results confirm the trends observed at higher temperatures, which is a strong decreasing *z*(U) and a weak decreasing *y*(Te2) with decreasing temperature in contrast to the increasing *z*(Te1), even for temperatures below 100 K, where generally the lattice dynamic effects are much less pronounced. This serves as an additional strong argument in favour of no structural change between RT and LT.

Table 5 ▸ shows the selected interatomic distances in UTe_2_ at 2.7 K compared with the data for 118 K from Stöwe (1996 ▸), both refined in space group *Immm*. As the lattice parameters and consequently the bond lengths at these two temperatures were determined in two independent experiments by different methods with different precision, a direct comparison between the absolute values would be questionable. However, calculated changes in the interatomic distances may be normalized by the relative shrinking of the crystal lattice. In our case the (U–U)*a* distance or the (Te2–Te2)*a* distances keep the lattice translation constant *a* and were used for such a normalization. This is justified by the fact that all lattice parameters have a similar relative change, as follows both from our results mentioned in the previous section and from (Stöwe, 1996 ▸). The column Change in Table 5 ▸ shows the relative change of the noted distance compared to the change of the lattice parameter *a* between the two temperatures. The values >1 denote a relative shortening, and values <1 a relative elongation of the noted distance comparing to the shrinking of the lattice parameter *a*. For example, (U–U)*c* is shortened by almost 60% more then (U–U)*a*, and (Te2–Te2)*b* in prism is effectively elongated by 30% more than (Te2–Te2)*a*. Thus, the main difference between the 118 K structure (similar to RT) and LT structure is a shift of the *z*(U) position resulting in the significant relative shortening of the (U–U)*c* distance, accompanied by a stretching of the U—Te1l bond as well as the (Te2—Te2)*b* length. Other distances do not change significantly. This behaviour further increases the anisotropy between the Te2 in prism and the Te1 in cap observed at RT (Burdett *et al.*, 1978 ▸). In Stöwe (1996 ▸), the possibility of formation of U–U bonding over the extended 5*f* wavefunctions is noted. It is worth mentioning that our LT structure result would strongly support such an U–U interaction within a biprism block along the *c* direction.

The first-coordination sphere polyhedron of the U atom should be analysed more carefully in order to identify the potential magnetic interaction pathways and its possible relevance for superconductivity. As shown in Fig. 2 ▸, U is surrounded by eight Te ions, forming a bicapped trigonal prism. Two such polyhedra share four Te2 ions, forming a rectangular face in the *ab* plane with periodicity *c*/2 and homogeneous bond distance U—Te2 and shortest distance between U atoms (U–U)*c*, form a building unit. The resulting fourfold capped biprisms are arranged along the *c* axis in the form of a body-centred lattice. Along the *a* axis, the biprisms share triangular faces and are stacked in chains with a periodicity of the lattice constant *a*, creating a dense U packing with second-longest distance between U atoms (U–U)*a*. Along the *b* axis, the biprisms lie in the periodicity of the *b* translation, relatively loosely, sharing only two Te1 corners. As shown by Burdett *et al.* (1978 ▸), such bicapped trigonal prismatic coordination results in strong electronic anisotropy between ligands in Te1 and Te2 sites. From the analysis of the electron localization function maxima, Stöwe *et al.* (1997 ▸) proposed to describe the charge distribution in UTe_2_ by the formula U^1.9+^Te^1.2−^Te^0.7−^ rather than by U^4+^[Te^2−^]_2_. Interestingly, a similar bicapped trigonal prism coordination of the U ion is present in superconducting UGe_2_. Although the average structure of the latter is different (space group *Cmmm*, No. 65) (Oikawa *et al.*, 1996 ▸), there are also fourfold capped biprisms forming a building unit. In UGe_2_, those blocks are more separated along the *c* direction than in UTe_2_ by an additional Ge layer. This leads to the clear in-plane magnetic anisotropy. Such anisotropic magnetization along the *a* direction, characteristic for FM UGe_2_, is also observed for UTe_2_ (Ran, Eckberg *et al.*, 2019 ▸). In the latter it is much weaker, as the ratio (U–U)*a*/(U–U)*c* = 1.108 is larger in comparison to UGe_2_ where it is only 1.04. Under applied pressure the ratio may change, leading to changes in the magnetic interaction paths, which are reflected in the observed FM superconductivity temperature-enhancement under applied pressure in UGe2. Pressure-dependent studies on UTe_2_ are currently under way (Ran, Kim *et al.*, 2019 ▸; Braithwaite *et al.*, 2019 ▸).

Neutron scattering is sensitive to the magnetic structure and is generally used to determine the direction and magnitude of the ordered magnetic moment. However, the sensitivity of the method with respect to weak magnetic moments is limited. If no additional magnetic Bragg reflections occur [*e.g.* in the case of FM or antiferromagnetic (AFM) structure with **k** = 0], the only information about magnetic order can be obtained from the fit of the magnetic structure model in the structural refinement. From magnetization measurements in UTe_2_, the negative Curie–Weiss temperature of 67 K and an effective magnetic moment of 3.2 µ_B_/U were reported (Noel & Troc, 1979 ▸), suggesting an AFM ordering. Note that the absence of any Bragg reflections with *h* + *k* + *l* = 2*n* + 1 as mentioned above, rules out the AFM structures with commensurate propagation vectors **k** (1,0,0), (0,1,0) and (0,0,1) and subsequently also (½,0,0), (0,½,0), (0,0,½). To prove this, a number of such half-indexed peaks as well as peaks with **k** = (½,½,0) were scanned at 1.7 K (lowest temperature in our cryostat). No evidence for the peaks ( 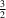 0 0), (0 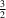 0), ( 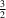 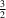 0), (2 ½ 0) and (2 1 ½) up to a level of < 5 × 10^−3^ to the main nuclear peaks was observed. Also a number of Q scans along [*hh*0] and [ 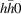 ] directions between 0.4 < *h* < 2.1 were performed at the same temperature, but do not show the presence of any incommensurate magnetic reflection within the limits of the instrument sensitivity. In order to check whether any AFM *k* = 0 or FM ordering would be compatible with our neutron diffraction data, we performed a number of refinements assuming an ordered magnetic moment on the U site with different FM and AFM subgroups starting from the paramagnetic space group *Immm* (Petricek *et al.*, 2014 ▸). All refinement attempts using magnetic symmetry subgroups led to worse-fit reliability factors than the structural fit without any static magnetic order. Certainly our results do not exclude the existence of a weak magnetic order, but rather determine the upper limit for such an ordered moment to be lower than 0.7 (2) μ_B_/U.

## 4. Summary

Our single-crystal neutron diffraction results are consistent with previously measured electrical resistivity, magnetization and specific heat data over a wide temperature range (Ran, Eckberg *et al.*, 2019 ▸). All evidence points to the absence of both structural and magnetic phase transitions in UTe_2_ between room temperature and 2.7 K, in contrast to previous reports (Stöwe *et al.*, 1997 ▸; Stöwe, 1996 ▸). Instead, the large temperature dependence of the transport properties and the magnetic anisotropy are the result of strongly interacting uranium-based *f*-states. This fact is reflected in the observed relatively large linear thermal expansion coefficient and a pronounced change in the *z* coordinate of the U position as well as in the (U–U)*c* distance, which was observed even at very low temperatures, where the lattice dynamics are usually damped. Crucially, there is no static magnetic order in UTe_2_ in the normal state, which makes this superconductor qualitatively different from ferromagnetic URhGe, UCoGe and UGe_2_ (Aoki *et al.*, 2019 ▸) despite the similar anisotropy in the superconducting upper critical fields and certain similarities in the crystal structure. Our new diffraction data also support the picture of UTe_2_ as a quantum critical ferromagnet, as there is no evidence for antiferromagnetic order that could produce the unusual field-temperature scaling of the magnetic susceptibility reported earlier (Ran, Eckberg *et al.*, 2019 ▸). The novel emergence of spin-triplet superconductivity from a paramagnetic normal state characterized by strong ferromagnetic spin fluctuations calls for focused theoretical attention. Detailed structural parameters for UTe_2_ at LT are reported for the first time and provide fundamental input for further experimental investigations and theoretical modelling.

## Supplementary Material

Crystal structure: contains datablock(s) I. DOI: 10.1107/S2052520619016950/ra5072sup1.cif

Structure factors: contains datablock(s) global, I. DOI: 10.1107/S2052520619016950/ra5072Isup2.hkl

CCDC reference: 1972889

## Figures and Tables

**Table 1:** Single-crystal neutron diffraction experimental data

Crystal data	
Chemical formula	UTe_2_
Relative molar mass	493.23
Cell setting, space group	Orthorhombic, *Immm*
*T* (K)	2.7
*a*, *b*, *c* (Å)	4.123 (5), 6.086 (9), 13.812 (17)
*V* (Å^3^)	346.6 (7)
*Z*	4
*D_x_* (Mg m^−3^)	9.1993
μ (mm^−1^)	0.0095
Crystal form, colour	Plate-like, black
Crystal size (mm)	3 × 3 × 1
	
Data collection	
Diffractometer	Normal-beam diffractometer POLI
Radiation source	Nuclear reactor
Monochromator	Cu(220)
Radiation type	Constant wavelength neutron
Wavelength (Å)	0.904 (1)
Data collection method	ω-scans
[sin θ/λ]_max_ (Å^−1^)	0.63
Range of *h*, *k*, *l*	−5→*h*→5, −6 →*k*→7, −9→*l*→13
No. of measured reflections	327
No. of observed reflections with *I* > 10σ(*I*)	298
No. of independent reflections with *I* > 10σ(*I*)	133
*R*_int_ (%)	1.62

**Table 2:** The maximal subgroups of the parent space group *Immm*

Subgroup type	Space group (No.)	Lattice type
*Translationengleiche*	*Imm*2 (44)	Body-centred orthorhombic
	*I*222 (23)	Body-centred orthorhombic
	*C*12/*m*1 (12)	Monoclinic
*Klassengleiche*	*Pmmn* (59)	Primitive orthorhombic
	*Pnnm* (58)	Primitive orthorhombic
	*Pnnn* (48)	Primitive orthorhombic
	*Pmmm* (47)	Primitive orthorhombic

**Table 3:** Refinement results of single-crystal neutron diffraction data for different symmetry allowed structural models using isotropic displacement parameters only, for better comparison

	Space group		
Fit result	*Imm*2	*I*222	*Immm*
No. of parameters	13	8	8
*R* factor (%)	1.51	1.52	1.52
*wR* factor (%)	2.10	2.10	2.10
Goodness-of-fit	1.53	1.51	1.51

**Table 4:** Fractional atomic coordinates, isotropic and anisotropic atomic displacement parameters for UTe_2_ At 2.7 K and refined in the orthorhombic space group *Immm* according to the present single-crystal neutron diffraction data. In this model *U*_12_, *U*_13_ and *U*_23_ are zero by symmetry.

Atom	Wyckoff position	*x*	*y*	*z*	*U*_11_ (Å^2^)	*U*_22_ (Å^2^)	*U*_33_ (Å^2^)	*U*_iso_ (Å^2^)
U	4*i*	0.00000	0.00000	0.13473 (6)	0.0021 (2)	0.0019 (3)	0.0014 (5)	0.0018 (2)
Te1	4*j*	0.50000	0.00000	0.29799 (10)	0.0033 (3)	0.0035 (4)	0.0034 (8)	0.0033 (3)
Te2	4*h*	0.00000	0.25062 (13)	0.50000	0.0035 (3)	0.0039 (4)	0.0031 (8)	0.0035 (3)

**Table 5:** Comparison between selected interatomic distances (shorter than 4.5 Å) at 2.7 K and 118 K from Stöwe (1996 ▸) The definition and an interpretation of the column Change are given in the text. Suffix s indicates short distance and suffix l indicates long distance.

Distance	2.7 K	118 K	Change
U–Te coordination polyhedra			
U–Te1s	3.0553 (12)	3.0778 (4)	1.08
U–Te1l	3.1817 (5)	3.1990 (3)	0.80
U–Te2 prism	3.1648 (6)	3.1898 (3)	1.15
U–U distances			
(U–U)*c* in the biprisms	3.7218 (17)	3.7630 (6)	1.61
(U–U)*a* chain of biprisms	4.123 (1)	4.1512 (3)	1.00
Te–Te distances			
Te1–Te2 cap to prism	3.7896 (11)	3.8190 (4)	1.13
Te1–Te1 cap to prism	3.9073 (10)	3.9326 (4)	0.95
Te1–Te1 in prism	4.123 (1)	4.1512 (3)	1.00
Te1–Te2 in prism	4.3868 (14)	4.4252 (6)	1.28
(Te2–Te2)*b* in prism	3.0355 (11)	3.050 (1)	0.70
(Te2–Te2)*b* prism–prism	3.0505 (11)	3.069 (1)	0.89
Te2–Te2*a* in prism	4.123 (1)	4.1512 (3)	1.00
